# Morphological characterization of Sussex cattle at Huntersvlei farm, Free State Province, South Africa

**DOI:** 10.1371/journal.pone.0292088

**Published:** 2023-09-26

**Authors:** Lubabalo Bila, Dikeledi Petunia Malatji, Thobela Louis Tyasi

**Affiliations:** 1 Potchefstroom College of Agriculture, Department of Animal Production, Potchefstroom, South Africa; 2 Department of Agriculture and Animal Health, College of Agriculture and Environmental Sciences, University of South Africa, Pretoria, South Africa; 3 School of Agricultural and Environmental Sciences, Department of Agricultural Economics and Animal Production, University of Limpopo, Limpopo, South Africa; PLOS (Public Library of Science), UNITED KINGDOM

## Abstract

Sussex cattle breed is characterized by their distinctive solid red coat colour and white tail switch. Sussex cattle are known for being easy to handle and manage, making them an ideal choice for cattle farmers. The phenotypic characterization of this cattle breed in South Africa is unknown. Hence, the objective of this study was to characterize the morphological structure, phenotypic and body indices traits of Sussex cattle in South Africa at Huntersvlei farm, Frere State province of South Africa. One hundred and one weaners (n = 101) between 6 and 8 months old (female = 57 and male = 44) and fifty yearlings between 12 and 15 months old (female = 15 and male = 35) were used in this study. Body weight at weaning, yearling and linear body measurements such as head length (HL), head width (HW), ear length (EL), ear width (EW), sternum height (SH), withers height (WH), heart girth (HG), hip height (HH), body length (BL), rump length (RL), and rump width (RW) were measured. Moreover, the animals were assessed for coat colour and horn presence. Descriptive statistics, Pearson’s correlation and Principal Component Analysis (PCA) were used to describe the Sussex cattle breed. The results indicated that male Sussex cattle had highly significant (p < 0.01) mean numeric values for the BW and morphometric traits. The results further showed that Sussex cattle had highly significant (p < 0.01) increase for the BW and morphometric traits as age advances in all sexes. Interaction effect of sex and age showed a highly significant (p < 0.01) effect with BW and measured morphometric traits, while moderately significant (p < 0.05) with EW. Male Sussex cattle showed highly significant (p < 0.01) higher mean numeric values for the body index (BI), length index (LI) and compact index (CI) indices. While female Sussex animals showed highly significant (p < 0.01) mean numeric values for area index (AI) and proportionality (PR). Body weight showed a positive highly significant (p < 0.01) correlation with the measured morphometric traits except for the moderate significant (p < 0.05) correlation with EL. Coat colour traits ranged from 15 (9.93%), 103 (68.21%) to 33 (21.85%) for light, moderate and dark colours, respectively. While horn presence traits ranged from 48 (31.79%), 42 (27.81%) to 61 (40.40%) for polled, scur and horned respectively. The PCA results extracted only two components in both sexes of the animals. The morphological variations obtained in this study could be complemented by performance data and molecular markers of single nucleotide polymorphism (SNP) to guide the overall breed characterization, conservation and development of appropriate breeding and selection strategies.

## Introduction

Livestock plays an important role in the social, economic and cultural stability of rural households in many parts of the world [1]. MASA [2] reported that livestock contributes meaningfully in many livelihoods by providing milk, meat, draught power and transport. Cattle are one of the animals that can be found in almost all the countries in the world, including South Africa with many species diversities. Sussex cattle breed is one of the oldest and pure breeds of English used for meat production in the world and it originates from the Weald of Sussex, Surrey and Kent in Southeastern England [13]. Characterization of cattle breeds is the first approach to the sustainable use its animal genetic resources [3]. Morphometric is a quantitative analysis practice (body dimensions) that includes the size and shape of an animal [4]. The morphometric data is essentially used to investigate an animal’s anatomic structure, productivity, growth rate, and performance quality of livestock [4]. Morphological measurements have been traditionally used for the characterization of cattle breeds by many scientists around the world [3]. Furthermore, morphometric measurements are carried out directly by measuring an animal’s body traits using a measuring tape, measuring stick or ruler, regarding the bony prominence (tuberosity or processus) starting from animal height, heart girth, body length, chest circumference, hip height and rump length [4]. Morphometric indices are associated with linear body measures used to define animal proportions and size and could be generated using linear body measures. These indices are a mixture of many linear body measures used to analyze animal breed type, weight, and function and help breeders choose appropriate breeding stock in their existing production system [5]. Such indices give empirical values based on morphometric features and are limited in using single measurements [6]. On the other hand, phenotypic information is a foundation for the establishment of further characterization, conservation and selection strategies [3]. Furthermore, the on-farm phenotypic characterization of cattle breeds is a primary and low-cost animal genetic resource characterization as compared to the on-station characterization [7]. Ahmed et al. [8] indicated that genetic relationships between livestock breeds can be observed by discriminating the breeds either based on genomic information and morphological measurements. Although using genomic information is more accurate for breed differentiation, discrimination of breed and morphometric measurement can be a first step to observing similarity among livestock breeds [8]. Several studies have been conducted in morphometric measurements to differentiate breeds of cattle in India [9] Nigeria [10] Ethiopia [11] and Indonesia [12]. However, to the deepest of our knowledge, there is no literature documented on the on-farm morphological characterization of Sussex cattle in South Africa. Hence, the objective of this study was to carry out a morphometric characterization of Sussex cattle in South Africa using body morphological measurements and body indices. This study will help cattle farmers to select the best morphological traits that might be used for improving cattle breeds during the breeding season.

## Materials and methods

### Ethics statement

The experimental procedures were conducted following the University of South Africa (UNISA) Ethics code for the use of live animals in research, ethics reference number: 2022/CAES_AREC/171.

### Study site

This study was conducted at Huntersvlei farm also known as Rhys Evans Group (RE) in the Free State Province, South Africa. The farm is situated in Viljoenskroon, Fezile Dabi municipality; the site, temperatures, latitudes, longitude, and rainfall of the study area similar as described by Bila [13]. Huntersvlei farm is one of the oldest and leading Sussex cattle stud herd in South Africa.

### Animal management

All the animals used in the study were exposed to a traditional management grazing system which allows animals to freely graze in the camps during the day and afternoon. Fresh clean water was always available in the camps. Animals received a routine inspection and dipping for herd health management purposes. The linear body measurements were taken while the animal was in a standing position with head raised up and weighed on all four feet. A functional handling facility with a crowding pen, working crush and head clamp was used for handling the animals to minimize movement during the measuring process.

### Data collection

Morphometric traits and live body weight at two different stages were taken. The first stage was at weaning age in one hundred and one (n = 101) (female = 57 and male = 44) South African Sussex weaner animals between 6 and 8 months old. Second stage yearling age is 50 (female = 15 and male = 35) South African Sussex yearling animals are between 12 and 15 months old. The animals used in the study at weaning age were between six to fifteen months old. The live body weight at the two stages was measured using a balance weighing scale whereas linear body measurements were measured using a measuring tape calibrated in centimetres (cm). The body weight at weaning, yearling and morphometric traits, namely head length (HL), head width (HW), ear length (EL), ear width (EW), sternum height (SH), withers height (WH), heart girth (HG), hip height (HH), body length (BL), rump length (RL), and rump width (RW) were measured following the guideline defined by Lomillos and Alonso [14], Tyasi et al. [15], Bila et al. [16] and Hlokoe et al. [17] (Table 1).

**Table 1:** Morphometric traits and their description.

Traits	Description
Head length (HL)	Measured from the temple of the head to the tip of the horn.
Head width (HW)	Measured as the space between the edges of the head.
Ear length (EL)	Measured as the space from the position of attachment to the tip of the ear.
Ear width (EW)	Measured as the distance between the middle of the top and bottom edge of the ear.
Sternum height (SH)	Measured as the vertical position from the lower tip of the sternum to the ground as the animal standing.
Withers height (WH)	Measured as a vertical position between the ground and the apex of the tourniquet, immediately behind the hump, on the top of the scapula.
Heart girth (HG)	Measured as the circumference of the chest.
Hip height (HH)	Measured as the space from the surface of a platform to the rump
Body length (BL)	Measured as the space from the highest position of shoulders to the pin bone.
Rump length (RL)	Measured as the distance from the hip bone to edge of the pin bone.
Rump width (RW)	Measured as the position between two tuber coxae.

To prevent individual variations in measurements, only one individual was taking the body weight and morphometric traits. The horn presence was assessed using three point scale (1 = polled; 2 = scur and 3 = horned) following the guidelines reported by Grobler et al. [18]. Moreover, the animals were assessed using a three-point scale (1 = light, 2 = moderate and 3 = dark) (Table 2).

**Table 2:** Qualitative traits and their description.

Traits	Trait category	Description
	Polled	Animal without horns.
Horn presence	Scur	Animal with incompletely developed horn growth that are not attached to the skull.
	Horned	Animal that has horns attached to the skull, blood vessels and nerves.
	Light	Light red colour.
Coat colour	Moderate	Moderate red colour.
	Dark	Dark red colour.

Table 3 below shows how the seven body indices were computed from morphometric traits as described by Birara et al. [19].

**Table 3:** Procedures used for calculating body indices from the recorded morphometric data.

Body indices	Formula used to calculate the indices
Body index (BI)	Body length / Heart girth * 100
Length index (LI)	Body length / Withers height
Pelvic index (PI)	Rump width / Rump length * 100
Area index (AI)	Withers height * Body length
Thoracic development index (TDI)	Heart girth / Withers height
Compact index (CI)	Weight / Withers height * 100
Proportionality (PR)	Withers height / Body length * 100

### Statistical analysis

The Statistical Analysis System [20] version 94.0 was used for data analysis. Procedure of means (PROC MEANS) was used for descriptive statistics of quantitative traits while procedure of frequency (PROC FREQ) was used for descriptive statistics of qualitative traits. Procedure of analysis of variance (PROC ANOVA) was used to observe the significant difference on measured traits, while chi-square (χ^2^) test was used to find the significant difference on qualitative traits. Procedure of correlation (PROC CORR) was used to determine Pearson’s correlation matrix. These analyses were then followed by a principal component analysis (PCA) to reduce the dimensionality of the morphometric data using the procedure of principal component (PROC PRINCOMP and PROC FACTOR). Furthermore, to examine the morphometric traits and body indices that have the most discriminating power the stepwise discriminant analysis was applied using the STEPDISC procedure (PROC STEPDISC). The significant difference was observed at P<0.05 and highly significant at P<0.01.

## Results

### Descriptive statistics

The overall summary of the collected data (Table 4) discovered that the coefficient of variation of the measured traits ranged from 7.47% to 52.00%.

**Table 4:** Average live body weight (kg) and morphometric traits (cm) (mean) of South African Sussex cattle in Huntersvlei farm.

		Measured traits												
Effect and level	N	CBW	BW	HL	HW	EL	EW	SH	WH	HG	HH	BL	RL	RW
Overall	151	31.48	301.57	41.58	15.28	13.95	9.95	62.92	114.05	160.48	120.61	113.22	41.97	35.48
CV (%)	151	52.00	30.28	7.47	24.87	7.80	9.90	9.54	9.71	11.64	9.55	15.71	12.24	16.45
**Sex**														
Male	79	38.11	344.27	43.24	16.56	14.34	9.95	64.84	119.65	168.59	125.51	142.73	43.95	37.27
Female	72	24.19	254.722	39.75	13.89	13.51	9.94	60.82	107.92	151.58	115.23	122.78	39.79	33.51
P-value		0.00**	0.00**	0.00**	0.00**	0.00**	0.98^ns^	0.00**	0.00**	0.00**	0.00**	0.00**	0.00**	0.00**
**Age**														
6 to 8 Months	101	28.12	250.62	40.38	12.76	13.74	9.81	59.45	107.51	151.24	113.68	120.89	39.39	32.32
12 to 15 Months	50	38.26	404.48	44.00	20.38	14.36	10.22	69.92	127.26	179.16	134.60	158.12	47.18	41.86
P-value		0.00**	0.00**	0.00**	0.00**	0.00**	0.02*	0.00**	0.00**	0.00**	0.00**	0.00**	0.00**	0.00**
**Sex by Age**														
Male, 6 to 8 Months	44	36.36	269.27	41.75	13.05	14.20	9.69	60.34	111.52	155.93	117.00	125.32	40.09	32.82
Female, 6 to 8 Months	57	21.75	236.23	39.32	12.54	13.39	9.91	58.77	104.42	147.61	111.12	117.47	38.84	31.93
Male, 12 to 15 Months	35	40.31	438.54	45.11	20.97	14.51	10.29	70.49	129.86	184.51	136.20	164.63	48.80	42.86
Female, 12 to 15 Months	15	33.46	325.00	41.40	9.00	14.00	10.07	68.60	121.20	166.67	130.87	142.93	48.80	39.53
P-value		0.00**	0.00**	0.00**	0.00**	0.00**	0.05*	0.00**	0.00**	0.00**	0.00**	0.00**	0.00**	0.00**

**Correlation significant (P < 0.01)

*Correlation significant (P < 0.05), ns: not significant, CBW: calf birth weight, BW: birth weight, HL: head length, HW: head width EL: ear length, EW: ear width, SH: sternum height, WH: withers height, HG: heart girth, HH: hip height, BL: body length, RL: rump length, RW: rump width.

### Sex effect on body weight and morphometric traits

Means and standard deviation for the sex effect on body weight and morphometric traits is given in Table 4. The male Sussex cattle at Huntersvlei farm had a higher highly significant (p < 0.01) mean numeric values for the body weight and morphometric traits while ear width had an insignificant (p > 0.05) mean value.

### Age effect on body weight and morphometric traits

Table 4 shows the age effect on body and morphometric traits on Sussex cattle at Huntersvlei farm. The body weight and morphometric traits showed a highly significant (p < 0.01) mean values on age groups while EW showed a moderately significant (p < 0.05) effect on mean values. These findings indicate that there was a significant increase in body weight and morphometric traits as the age increases from weaners to yearling.

### Sex and age interaction effect on body weight and morphometric traits

Table 4 shows the sex and age effect on body and morphometric traits on Sussex cattle at Huntersvlei farm. The interaction effect of sex and age showed a highly significant (p < 0.01) effect with BW and all the measured morphometric traits, while moderately significant (p < 0.05) with EW. In general, male animals showed a higher BW and mean values for the measured morphometric traits across all ages. While female animals had lower mean numeric values for BW and measured morphometric traits in all ages, this may be resulted by physiological induces (hormonal secretions) and other activities in various sexes.

### Body indices

#### Descriptive statistics

The overall summary of the collected data (Table 5) discovered that the coefficient of variation of the calculated body indices traits ranged from 6.17% to 70.04%.

**Table 5:** Body indices (Mean) of South African Sussex cattle in Huntersvlei farm.

		Body indices						
Effect and level	N	BI	LI	PI	AI	TDI	CI	PR
Overall	151	83.05	1.16	84.44	0.86	1.41	260.05	59.98
CV (%)	151	11.17	7.59	10.25	7.58	6.17	20.88	70.04
**Sex**								
Male	79	84.97	1.19	84.73	0.85	1.41	283.37	50.03
Female	72	80.94	1.14	84.13	0.88	1.41	234.46	70.89
P-value		0.00**	0.00**	0.67^ns^	0.00**	0.86^ns^	0.00**	0.00**
**Age**								
6 to 8 Months	101	80.32	1.12	82.19	0.89	1.41	232.42	89.27
12 to 15 Months	50	88.54	1.24	89.00	0.81	1.41	315.87	0.05
P-value		0.00**	0.00**	0.00**	0.00**	0.99^ns^	0.00**	0.00**
**Sex by Age**								
Male, 6 to 8 Months	44	81.24	1.12	82.12	0.89	1.39	241.15	89.20
Female, 6 to 8 Months	57	79.64	1.12	82.24	0.89	1.41	225.68	89.32
Male, 12 to 15 Months	35	89.67	1.27	88.00	0.79	1.42	336.45	0.79
Female, 12 to 15 Months	15	85.88	1.18	91.32	0.85	1.37	267.86	0.85
P-value		0.00**	0.00**	0.00**	0.00**	0.30^ns^	0.00**	0.00**

**Correlation significant (*P* < 0.01)

*Correlation significant (*P* < 0.05), ns: not significant, BI: body index, LI: Length index, PI: Pelvic index, AI: Area index, TDI: Thoracic development index CI: Compact index, PR: Proportionality, N: number.

#### Sex effect on body indices

Means and standard deviation for the overall sex effect on body indices is given in Table 5. The male Sussex cattle at Huntersvlei farm showed a highly significant (p < 0.01) higher mean numeric values for the BI, LI and CI indices. While female Sussex animals at Huntersvlei farm showed a highly significant (p < 0.01) higher mean number values for AI and PR Indice. Lastly, these results showed an insignificant (p > 0.05) mean numeric values between PI and TDI indices.

#### Age effect on body indices

Table 5 shows the age effect on body indices of Sussex cattle at Huntersvlei farm. The yearling animals showed a highly significant (p < 0.01) higher mean numeric values for BI, LI, PI and CI body indices. While weaners showed a highly significant (p < 0.01) higher mean numeric values for AI and PI body indices. Furthermore, the TDI had an insignificant (p > 0.05) mean numeric values across ages.

#### Sex and age interaction effect on body indices

Table 5 shows the sex and age effect on body indices of Sussex cattle at Huntersvlei farm. The results showed that the interaction between sex and age had a highly significant (p < 0.01) in the calculated body indices except TDI (p > 0.05) as shown in Table 5.

### Correlation between body weight and morphometric traits

Table 6 shows Pearsons correlation coefficient between body weight and morphometric traits of males and females Sussex cattle at Huntersvlie farm. In males, the CBW showed insignificant (p > 0.05) correlation with all the morphometric traits except EL that had a negative moderate significant (p < 0.05) correlation (Table 6). BW showed a positive highly significant (p < 0.01) correlation with all the morphological traits except moderate significant (p < 0.05) correlated with EL. In female, the CBW showed a positive highly significant (p < 0.01) correlation with BW, SH, WH, HG, HH, BL, RL and RW while positive moderate significant (p < 0.05) with HW. CBW showed an insignificant (p > 0.05) correlation with HL, EL and EW. BW showed a positive highly significant (p < 0.01) correlation with HL, HW, SH, WH, HG, HH, BL, RL and RW, while positive moderate significant (p < 0.05) correlated with EL and insignificant (p > 0.05) correlated EW.

**Table 6:** Correlation coefficients of measured traits in males below the diagonal and females above the diagonal.

Traits	CBW	BW	HL	HW	EL	EW	SH	WH	HG	HH	BL	RL	RW
CBW	1	0.42**	0.22^ns^	0.27*	0.03^ns^	0.02^ns^	0.40**	0.43**	0.45**	0.38*	0.43**	0.35**	0.32**
BW	0.15^ns^	1	0.75**	0.73**	0.26*	0.21^ns^	0.65**	0.81**	0.88**	0.82**	0.73**	0.63**	0.77**
HL	0.09^ns^	0.77**	1	0.42**	0.27*	0.31**	0.44****	0.58**	0.68**	0.60**	0.50**	0.50**	0.63**
HW	0.19^ns^	0.91**	0.67**	1	0.24*	0.11^ns^	0.00**	0.84**	0.72**	0.84**	0.78**	0.54**	0.71**
EL	-0.24*	0.26*	0.31**	0.18^ns^	1	0.12^ns^	0.34**	0.39**	0.33**	0.41**	0.38**	0.32**	0.32**
EW	0.11^ns^	0.21^ns^	0.12^ns^	0.23*	0.09^ns^	1	0.17^ns^	0.17^ns^	0.14^ns^	0.09^ns^	0.11^ns^	0.00^ns^	0.11^ns^
SH	0.17^ns^	0.78**	0.58**	0.82**	0.09^ns^	0.16^ns^	1	0.81**	0.69**	0.83**	0.76**	0.64**	0.72**
WH	0.17^ns^	0.94**	0.72**	0.91**	0.20^ns^	0.23*	0.81**	1	0.85**	0.93**	0.85**	0.68**	0.79**
HG	0.07^ns^	0.83**	0.55**	0.76**	0.19^ns^	0.21^ns^	0.72**	0.79**	1	0.86**	0.75**	0.71**	0.80**
HH	0.17^ns^	0.95**	0.74**	0.92**	0.26*	0.25*	0.80**	0.96**	0.78**	1	0.86**	0.71**	0.84**
BL	0.20^ns^	0.94**	0.69**	0.92**	0.23*	0.24*	0.81**	0.92**	0.76**	0.93**	1	0.65**	0.76**
RL	0.19^ns^	0.83**	0.66**	0.85**	0.18^ns^	0.21^ns^	0.74**	0.81**	0.71**	0.81**	0.85**	1	0.72**
RW	0.15^ns^	0.83**	0.63**	0.87**	0.20^ns^	0.10^ns^	0.77**	0.79**	0.69**	0.81**	0.81**	0.80**	1

**Correlation significant (*P* < 0.01)

*Correlation significant (*P* < 0.05), ns: not significant, CBW: calf birth weight, BW: birth weight, HL: head length, HW: head width EL: ear length, EW: ear width, SH: sternum height, WH: withers height, HG: heart girth, HH: hip height, BL: body length, RL: rump length, RW: rump width.

### Correlation between body indices traits

Table 7 shows Pearsons correlation coefficient between the calculated body indices of males and females Sussex cattle at Huntersvlie farm. In males, the BI showed a positive significant (p < 0.01) correlation with LI and CI while negatively highly significant (p < 0.01) correlated with AL, TDI and PR and insignificant (p > 0.05) correlated with PI (Table 7). LI showed a positive highly significant (p < 0.01) correlation with CI and negative highly significant (p < 0.01) correlation with AI and PR while insignificant (p > 0.05) with PI and TDI. PI showed insignificant (p > 0.05) correlation AI and TDI while positive highly significant (p < 0.01) correlated with CI and negative highly significant (p < 0.01) with PR. CI showed a negative highly significant (p < 0.01) correlation with PR.

**Table 7:** Correlation coefficients of body indices in males below the diagonal and females above the diagonal.

Indices	BI	LI	PI	AI	TDI	CI	PR
**BI**	1	0.84**	0.23*	-0.83**	-0.54**	0.04^ns^	-0.50**
**LI**	0.49**	1	0.25*	-1.00**	0.01^ns^	0.26*	-0.43**
**PI**	0.08^ns^	0.20^ns^	1	-0.25*	-0.03^ns^	0.46**	-0.47**
**AI**	-0.49**	-1.00**	-0.20^ns^	1	-0.01^ns^	-0.25*	0.43**
**TDI**	-0.75**	0.18^ns^	0.06^ns^	-0.18^ns^	1	0.33**	0.26*
**CI**	0.29**	0.77**	0.34**	-0.76**	0.28**	1	-0.49**
**PR**	-0.40**	-0.80**	-0.32**	0.80**	-0.12^ns^	-0.83**	1

**Correlation significant (*P* < 0.01)

*Correlation significant (*P* < 0.05), ns: not significant, BI: body index, LI: Length index, PI: Pelvic index, AI: Area index, TDI: Thoracic development index CI: Compact index, PR: Proportionality.

In females, the BI showed a positive highly significant (p < 0.01) correlation with LI, while moderately significant (p < 0.05) correlated with PI and insignificant (p > 0.05) correlated with CI (Table 7). PI showed a highly positive significant (p < 0.01) correlation with CI and negative highly significant (p < 0.01) correlation with AI while insignificant (p > 0.05) correlated with TDI. AI showed a positive highly significant (p < 0.01) correlation PR and negative moderate significant (p < 0.05) correlation with CI while insignificant (p > 0.05) with TDI. TDI showed a positive highly significant (p < 0.01) correlation with CI and positive moderate significant (p < 0.05) correlation with PR. Lastly, CI showed a negative highly significant (p < 0.01) correlation with PR (Table 7).

### Descriptive statistics of coat colour and horn presence

The overall summary of the collected data (Table 8) discovered that the coat colour traits ranged from 15 (9.93%), 103 (68.21%) to 33 (21.85%) for light, moderate and dark colour, respectively. While on the other hand, horn presence traits ranged from 48 (31.79%), 42 (27.81%) to 61 (40.40%) for polled, scur and horned respectively.

**Table 8:** Qualitative traits (N, %) of Sussex cattle in Huntersvlei farm.

		Coat colour			Horn presence			
Effect and level	N	Light	Moderate	Dark	Polled	Scur		Horned
Overall	151	15 (9.93)	103 (68.21)	33 (21.85)	48 (31.79)	42 (27.81)		61 (40.40)
**Sex**								
Male	79	6 (7.59)	48 (60.76)	25 (31.65)	16 (20.25)	10 (24.05)		44 (55.70)
Female	72	9 (12.50)	55 (76.39)	8 (11.11)	32 (44.44)	23 (31.94)		17 (23.61)
Significant		χ^2^ = 9.53, P-value = 0.01**			χ^2^ = 17.38, P-value = 0.00**			
**Age**								
6 to 8 Months	101	10 (9.90)	76 (75.25)	15 (14.85)	35 (34.65)	29 (28.71)		37 (36.63)
12 to 15 Months	50	5 (10.00)	27 (54.00)	18 (36.00)	13 (26.00)	13 (26)		24 (48)
χ^2^ P-value		χ^2^ = 9.06, P-value = 0.01**			χ^2^ = 1.95, P-value = 0.38^ns^			

**Correlation significant (*P* < 0.01), ns: not significant.

#### Sex effect on coat colour and horn presence

The frequency and percentage of coat colour and horn presence observed in males and females of Sussex cattle at Huntersvlei farm population are presented in Table 8. The results of the study showed that the coat colour in both male and female populations had a highly significant (p < 0.01) effect with males had a maximum incident of dark coat colour while female having a maximum incident moderate coat colour. While on the other hand horned presence showed a significant (p < 0.01) effect amongst the sexes with males having a maximum incident horned presence and females having a maximum incident of polledness.

#### Age effect on coat colour and horn presence

The frequency and percentage of coat colour and horn presence observed in males and females of Sussex cattle at Huntersvlei farm populations are presented in Table 8. The results of the study showed that coat colour in both weaners and yearling age had a highly significant (p < 0.01) effect with weaners having a maximum incident of moderate coat colour while yearling had a maximum incident of dark coat colour. Lastly, age had no effect (p>0.05) on horned presence.

**Fig 1:**
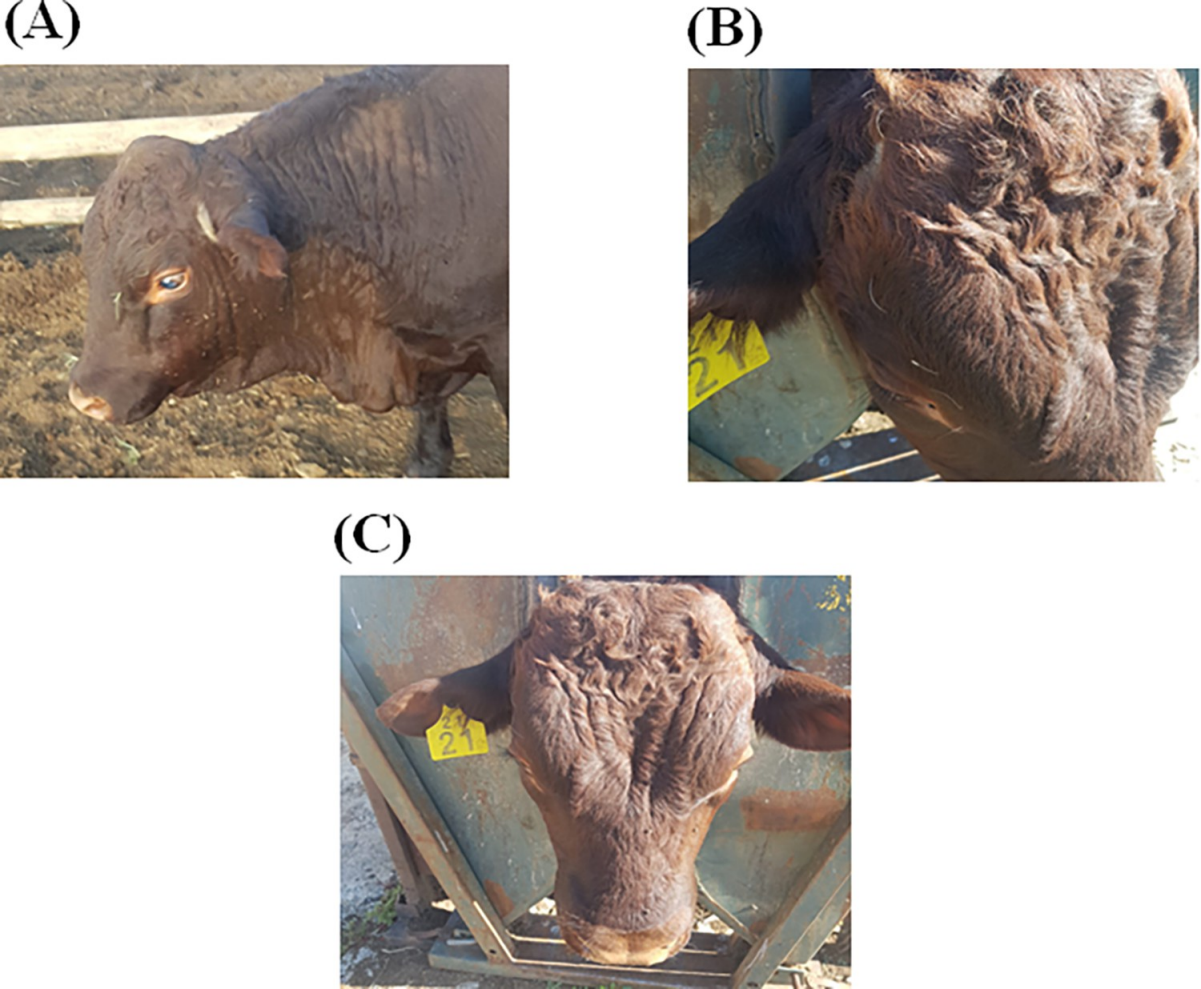
Morphological differences for polled, scur and horned in the South African Sussex cattle breed, illustrating differences in head shape [A] polled, [B] scur and [C] horned (Photographs by L Bila).

Fig 1 below shows the phenotypic identification of the horn presence polled, scur and horned phenotypes of Sussex cattle at Huntersvlie farm.

**Fig 2:**
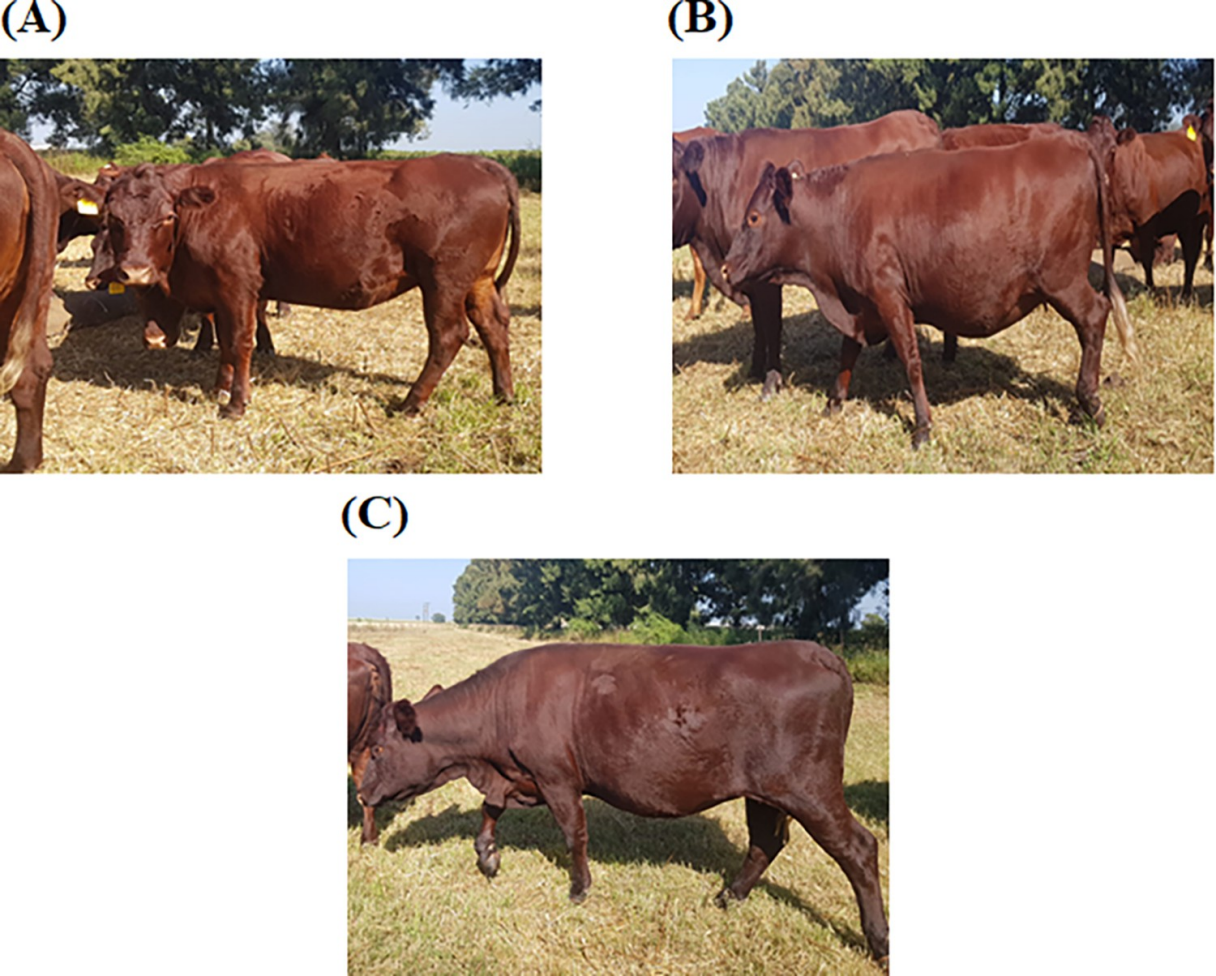
Morphological differences for light, moderate and dark red coat colour in the South African Sussex cattle breed, illustrating differences in coat colour [A] light, [B] moderate and [C] dark (Photographs by L Bila).

Fig 2 below shows the phenotypic identification of the coat colour light, moderate and dark phenotypes of Sussex cattle at Huntersvlie farm.

### Principal component analysis

**Fig 3:**
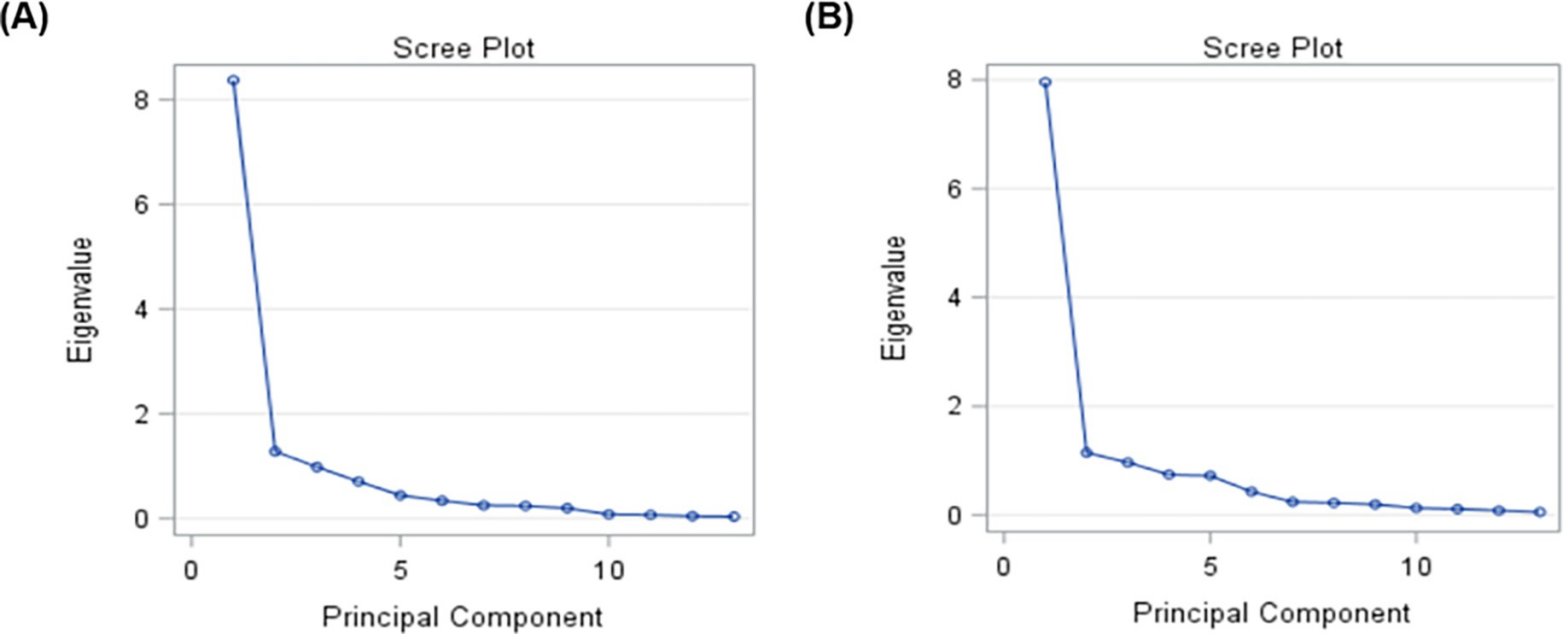
Scree plots showing a component number with Eigenvalue of both males (A) and females (B) Sussex cattle.

The predicted factor loading extracted by factor analysis, eigen-values and variation explained by each factor are presented in Table 9. The PCA results extracted only two components in both sexes of South African Sussex cattle. The extracted two components in the case of males of South African Sussex cattle accounted 74% of the variance. The first component explained 64% of variance and was presented by high loadings for BW, HW, WH, HH and BL. While, in the second factor accounted 10% of variance presenting high positive loadings for CBW. In female of South African Sussex cattle, the two extracted principal components contributed 70% of the variance in the data, thereby; the first component was enough to explain 61% of the total variance. Moreover, the second component explained 9% of variance and presented high positive loading for EW. The communality for males of South African Sussex cattle ranged from 0.08 for EW to 0.95 for BW. In females of South African Sussex cattle, the communality ranged from 0.25 for EL to 0.92 for HH. The communality after extraction gives the common variance that is shared amongst variables. Based on the eigen value higher than one, only two components for both sexes could be extracted based on the Scree plot (Fig 3).

**Table 9:** Eigenvalues, share of total variance along with loading and communalities of the morphometric traits of South African Sussex cattle.

Traits	Males			Females		
	PC1	PC2	Communality	PC1	PC2	Communality
CBW	0.19	0.79	0.66	0.47	-0.34	0.33
BW	0.97	-0.04	0.95	0.89	0.10	0.81
HL	0.77	-0.18	0.62	0.69	0.40	0.64
HW	0.96	0.06	0.92	0.84	-0.10	0.72
EL	0.25	-0.77	0.66	0.41	0.28	0.25
EW	0.25	0.12	0.08	0.18	0.85	0.76
SH	0.86	0.11	0.75	0.85	-0.08	0.73
WH	0.96	0.01	0.91	0.94	-0.03	0.89
HG	0.83	-0.04	0.70	0.92	0.00	0.85
HH	0.96	-0.02	0.93	0.96	-0.07	0.92
BL	0.96	0.03	0.91	0.89	-0.11	0.80
RL	0.89	0.05	0.80	0.78	-0.15	0.62
RW	0.88	0.00	0.77	0.89	-0.02	0.79
**Eigenvalue**	8.37	1.28		7.96	1.15	
**% of total variance**	64	10		61	9	

CBW: calf birth weight, BW: birth weight, HL: head length, HW: head width EL: ear length, EW: ear width, SH: sternum height, WH: withers height, HG: heart girth, HH: hip height, BL: body length, RL: rump length, RW: rump width, PC1: Principal component 1, PC2: Principal component 2.

### Canonical Discriminant Analysis (CDA)

#### Morphometric traits

The CDA for morphometric traits of South African Sussex cattle are presented in Table 10. Thirteen morphometric traits for both sexes were subjected to the STEPDISC procedure and six of them were identified as suitable discriminating variables of which HW and HL had the highest discriminating power to characterize the Sussex cattle breed at Huntersvlei farm. Wilks lambda test confirmed that all the six suitable traits had a highly significant (p < 0.001) contribution to discriminate the total into separate groups.

**Table 10:** Summary of stepwise discriminant analysis for selection of morphometric traits with discriminating power.

Step	Variables	Partial R-square	Pr > F	Wilks Lambda	Pr < Lamba	ASCC	Pr > ASCC
1	HW	0.90	< .00	0.10	< .00	0.90	< .00
2	HL	0.08	0.00	0.10	< .00	0.90	< .00
3	HH	0.07	0.00	0.09	< .00	0.91	< .00
4	SH	0.03	0.03	0.09	< .00	0.91	< .00
5	HG	0.04	0.01	0.08	< .00	0.92	< .00
6	RW	0.02	0.13	0.08	< .00	0.91	< .00

HW: head width, HL: head length, HH: hip height, SH: sternum height, HG: heart girth, RW: rump width and ASCC: average square canonical correlation.

#### Body indices traits

The CDA for body indices traits of South African Sussex cattle are presented in Table 11. Four body indices traits for both sexes were subjected to STEPDISC procedure and all of them were identified as suitable discriminating variables of which PR and AI had the highest discriminating power to characterize the Sussex cattle breed at Huntersvlei farm. Wilks lambda test confirmed that all the measured traits had a highly significant (p < 0.001) contribution to discriminate the total into separate groups.

**Table 11:** Summary of stepwise discriminant analysis for selection of body indices traits with discriminating power.

Step	Variables	Partial R-square	Pr > F	Wilks Lambda	Pr < Lamba	ASCC	Pr > ASCC
1	PR	0.99	< .00	0.01	< .00	0.99	< .00
2	AI	0.70	< .00	0.00	< .00	0.99	< .00
3	LI	0.64	< .00	0.00	< .00	0.99	< .00
4	CI	0.02	0.06	0.00	< .00	0.99	< .00

PR: Proportionality, AI: Area index, LI: Length index, CI: Compact index and ASCC: average square canonical correlation.

## Discussion

This study was exclusively based on specific phenotypic and morphometric traits of Sussex cattle at Huntersvlei farm in the Free State, Province, South Africa. Phenotypic variation of local animal resources shows a genetic diversity that may be worth conserving for future uses while better understanding of the external features assists to facilitate the implementation of conservation policies intended to ensure local resources survival [21]. Firstly, the study determined the descriptive statistics of the body weight and morphometric traits in both sexes (male and female) of the Sussex cattle at Huntersvlei farm at two different ages (weaning and yearling). The male animals showed higher significant mean numeric values compared to females for body weight and measured morphometric traits except for ear width. Furthermore, the results revealed that there was a positive significant effect on the body weight and measured morphometric traits in all age groups. These findings clearly indicate that body weight and morphometric traits increases proportionately with advancement in age. However, this circumstance is normal since the shape and size of animals change as the animal’s advances in age. These findings are similar with the reports made by Tyasi et al. [15] in Nguni cattle. However, Alonso et al. [22] indicated that the differences between genders might probably be due to increased selection pressure in the male as a result of the influence of the “trapio” that could be defined as a combination of physical qualities and presence necessary for the taurine celebrations, on the male’s economic value. Higher mean numeric values of the males in body weight and linear body measurements may be resulted by physiological induces (hormonal secretions) and other activities in various sexes [15]. Secondly, the study determined the descriptive statistics of the calculated body indices traits in both genders (male and female) of the Sussex cattle at Huntersvlei farm at two different ages (weaning and yearling). The results showed that there was a significant effect on sexes for the calculated indices except the insignificant thoracic development index and pelvic index. Moreover, the results showed that there was a significant effect on sex by age interaction in the calculated indices except the thoracic development index. The correlation results indicated that the male animals, body weight at all ages were highly correlated with hip height, withers height and body length while insignificant correlated with ear width. In female animals, the body weight at all ages was highly correlated with heart girth, hip height and withers height while insignificant correlated with ear width as well. The results of the present study are in agreement with the reports made by Tyasi et al. [15] who revealed that male Nguni cattle, linear body measurements (SH, HG and WH) had a significant positive correlation with live body weight. The results of the current study suggest that all the measured morphometric traits used in the study might be used for improvement of body weight in both male and female Sussex cattle at Huntersvlei farm at two different ages (weaning and yearling), except for ear width as it was insignificant associated with body weight. Maiwashe et al. [23] indicated that when traits are positively associated, it means that those traits are controlled by a similar gene. The correlation results between body indices revealed that male’s body index had a positive association with length index and compact index and a negative highly significant association with area index, thoracic development index and proportionality while insignificant with pelvic index. In females, the body index showed a positive association with length index, pelvic index and a negative highly significant association with area index, thoracic development index and proportionality while insignificant with compact index. The overall results for coat colour discovered that the coat colour traits ranged from 15 (9.93%), 103 (68.21%) to 33 (21.85%) for light, moderate and dark, respectively. While on the other hand horn presence traits ranged from 48 (31.79%), 42 (27.81%) to 61 (40.40%) for polled, scur and horned respectively.

Furthermore, the results of the study showed that the coat colour both in male and female populations had a highly significant effect with males showing a maximum incident of dark coat colour while females showing a maximum incident moderate coat colour. On the other hand, the horn presence showed a significant effect amongst the sexes with males showing a maximum occurrence of horn presence and females showing a maximum occurrence of polledness. Bila and Tyasi [24] showed that the morphological correlations do not take into account the cause influence between the traits or how much each trait contributes to the variation of body weight. Hence, the principal component analysis (PCA) was used to cluster related animals based on morphometric traits of Sussex cattle at Huntersvlei farm. The principal component analysis is a procedure depending on two or more variables with main function is to outline the fundamental structure among the analysed variables [25]. The PCA results extracted only two components in both sexes of South African Sussex cattle. The extracted two components in the case of males of South African Sussex cattle accounted 74% of the variance. The first component explained 64% of variance and was presented by high loadings for BW, HW, WH, HH and BL. While, in the second factor accounted 10% of variance presenting high positive loadings for CBW. In female of South African Sussex cattle, the two extracted principal components contributed 70% of the variance in the data, thereby; the first component was enough to explain 61% of the total variance. Moreover, the second component explained 9% of variance and presented high positive loading for EW.

## Conclusion

The importance of morphological characterization of cattle breed’s genetic resources cannot be emphasized. The present study is based on morphometric and certain phenotypic traits of Sussex cattle at Huntersvlei farm in South Africa. The study findings displayed the morphological and phenotypic variations between ages and genders of the Sussex cattle breed at Huntersvlei farm. It was found that the Sussex cattle breed in the study area have developed some diverse phenotypic traits such polled, scur and horns. Moreover, the Sussex cattle at Huntersvlei farm showed that the male animals had a much darker red brown colour in comparison to female animals. The correlation results shown that in male BW had highest positive association with HH, BL and WH and hence can be used as markers to predict BW using regression equations. The extracted principal components from different morphometric traits symbolize the general body size and shape of the Sussex cattle population at Huntersvlei farm. Stepwise canonical discriminant analysis showed that head width, head length, hip height, sternum height, heart girth and rump width are the suitable discriminating morphometric traits in the Sussex cattle breed. Furthermore, an investigation on the molecular characterization using molecular markers SNP will complement the results obtained from morphometric variation. Lastly these findings might be helpful in breed characterization, conservation and developing breeding and selection strategies.

## Supporting information

S1 File Raw data of quantitative traits. It is an Excel file with variables fully explained in column P and Q of the sheet.
(XLSX)

S2 File Raw data of qualitative traits. It is an Excel file with variables fully explained in column E, F and G of the sheet.
(XLSX)
